# Awareness and Attitude of Healthcare Workers to Cosmetic Surgery in Osogbo, Nigeria

**DOI:** 10.1155/2014/869567

**Published:** 2014-06-11

**Authors:** Opeyemi Adeniyi Adedeji, Ganiyu Oladiran Oseni, Peter Babatunde Olaitan

**Affiliations:** Department of Surgery, Ladoke Akintola University of Technology Teaching Hospital, Osogbo, Osun State, Nigeria

## Abstract

This study aimed at understanding the level of awareness and elucidates the attitude and disposition of healthcare workers to cosmetic surgery in Osogbo, Nigeria. A questionnaire-based survey was done at LAUTECH Teaching Hospital, Osogbo, in 2012. Questionnaires were administered to 213 workers and students in the hospital. These were then analysed using SPSS version 16.0 with frequencies, means, and so forth. Respondents were 33 doctors, 32 nurses, 79 medical students, 60 nursing students, 4 administrative staff, 1 pharmacist, and 4 ward maids. There is fair awareness about cosmetic surgery generally with 94.5% and its availability in Nigeria with 67.0%. A fewer proportion of the respondents (44.5%) were aware of the facility for cosmetic surgery in their locality. A large percentage (86.5%) favorably considers facilities outside Nigeria when making choice of facility to have cosmetic surgery done. 85.5% considered the information about cosmetic surgery reliable while 19.0% objected going for cosmetic surgery of their choice even if done free. Only 34.0% consider cosmetic surgery socially acceptable. Although the awareness of health workers about cosmetic surgery is high, their disposition to it is low. There is a need to increase the awareness in order to increase cosmetic surgery practice in Nigeria.

## 1. Introduction

The concern of people about their appearance is gradually on the increase both in the developed and the developing world; thus there is an increase in the number of cosmetic surgeries done annually. In the United States, for instance, 11.7 million cosmetic procedures were performed in 2007, with the vast majority being minimally invasive procedures [1]. Even though the rate of rise is not as high as that in the developing countries, the fact still remains that people are getting more concerned with their appearance especially with the increasing standard of living. In Asia, cosmetic surgery has become an accepted practice, and countries such as China and India have become Asia's biggest cosmetic surgery market [2]. It may not appear that plastic and reconstructive surgeries in children are of high priority in a country like Uganda. However, plastic surgery cases may constitute up to 20% of the surgical workload of a rural hospital in sub-Saharan Africa [3], and lack of surgical provision commits otherwise healthy individuals to lifelong disfigurement and functional impairment, as well as educational and social exclusion [4]. A number of factors may underscore the increase in the popularity of cosmetic surgery.

These include the growing importance of physical appearance in contemporary western culture [5, 6] which has served to normalize the pursuit of appearance-enhancing behaviours [7]. Higher disposable incomes among patients, advances in surgical procedures (particularly in terms of safety), and the lower cost of treatments have also served to reduce patient anxiety about cosmetic procedures [8].

In spite of the tremendous advancements in the field of plastic surgery, it seems that limited knowledge among the general public exists regarding the spectrum of plastic surgery especially cosmetic surgery. Has the medical community been well informed about cosmetic surgery? The knowledge of the general public about cosmetic plastic surgery will increase in proportion to the knowledge of the health workers who are likely to inform or misinform the general public about these surgical procedures. These people contribute considerably to the health care of the public.

This study is an attempt to establish the knowledge of the health community in a developing country on cosmetic surgery aspect of plastic surgery and their attitude to this subspecialty.

Understanding the attitude and perception of our medical colleagues and other health workers will be more vital than assessing the knowledge of the public. Students in the clinical years were also involved in this study as a means to have an idea of the awareness and disposition of these professionals to be and thus taking a peep into the future of cosmetic surgery in the country.

## 2. Materials and Method

We conducted a questionnaire-based study among a selected group of healthcare providers in a teaching hospital in Nigeria. These include doctors, nurses, medical students, nursing students, pharmacist, administrative workers, and ward maids/assistants to assess their knowledge, attitude, and perception of cosmetic surgery. A well-structured set of questionnaires was administered to this group of selected individuals, and responses were sought and analyzed. Only students that spent at least a year in the clinical part of their training were included. Two hundred and twenty questionnaires were administered.

The questionnaire has 3 parts; the first part assesses biodata and the second assesses the knowledge and awareness of cosmetic surgery while the third part assesses the attitude and disposition of the respondents to cosmetic surgery.

## 3. Results

Two hundred and thirteen (96.8%) responded to the questionnaires. Seventy-eight (36.6%) of them were males while 135 (63.4%) of them were females. The age range 18–30 years constituted the largest number of the respondents with 176 (82.6%) followed by 31–40 with 13.1% (Table 1).

Seventy-nine (37.1%) of the respondents were medical students, 60 (28.2%) were nursing students, 33 (15.5%) were doctors, 32 (15.0%) were nurses, 1 (0.5%) was a pharmacist, and 4 (1.9%) were ward assistants and administrative staff, respectively. Two hundred and one (94.4%) of them were aware of cosmetic surgery, and they were informed of cosmetic surgery by different means including television (106 (49.8%)) and radio (20 (9.4%)). Others were informed by friends, 33 (15.5%), posters, 21 (9.9%), medical consultation, 73 (34.3%), medical books, 33 (15.5%), newspaper and magazine, 1 (0.5%), and Internet, 11 (5.2%). One hundred and eighty-three (85.9%) of the respondents consider their sources of information about cosmetic plastic surgery as reliable while 13 (6.1%) did not think the sources were reliable and 11 (5.2%) were not sure their sources of information were reliable.

**Figure 1:**
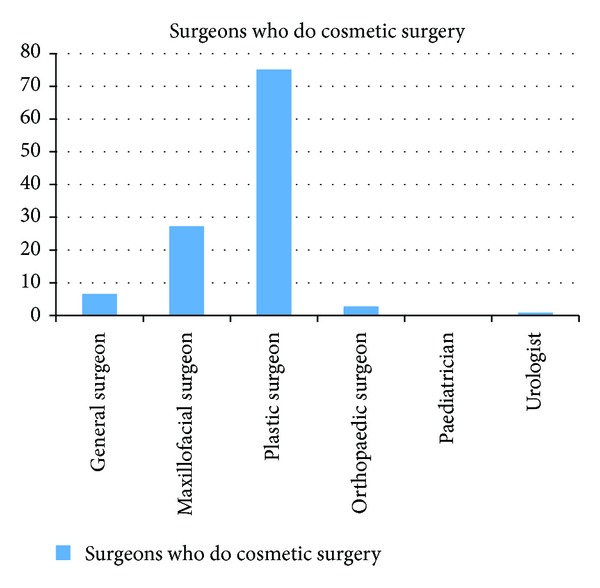
Surgeons suggested by respondents as involved in cosmetic surgery.

When asked “which surgeons do cosmetic surgery?” the responses showed that 58 (27.2%) considered that maxillofacial surgeons do cosmetic procedures and 160 (75.1%) agreed plastic surgeons do cosmetic procedures while 49 (23.1%) do not consider that plastic surgeons do cosmetic surgery. Other surgeons considered to be involved with cosmetic surgery are as shown in Figure 1.

**Figure 2:**
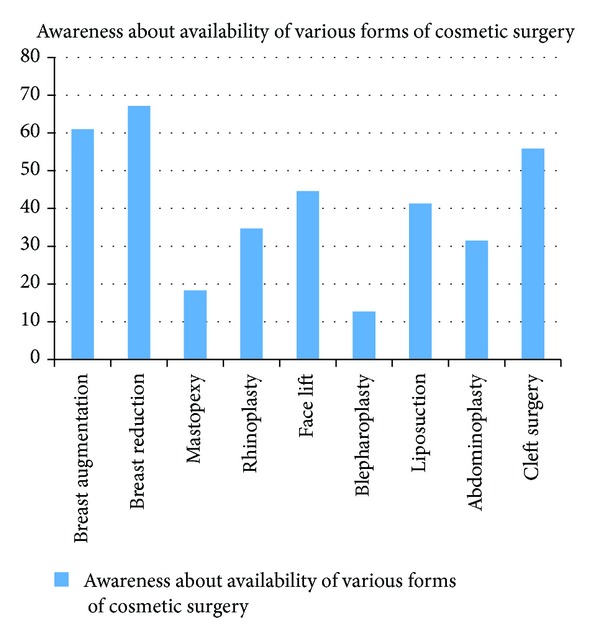
Awareness of respondents about cosmetic surgery.

**Figure 3:**
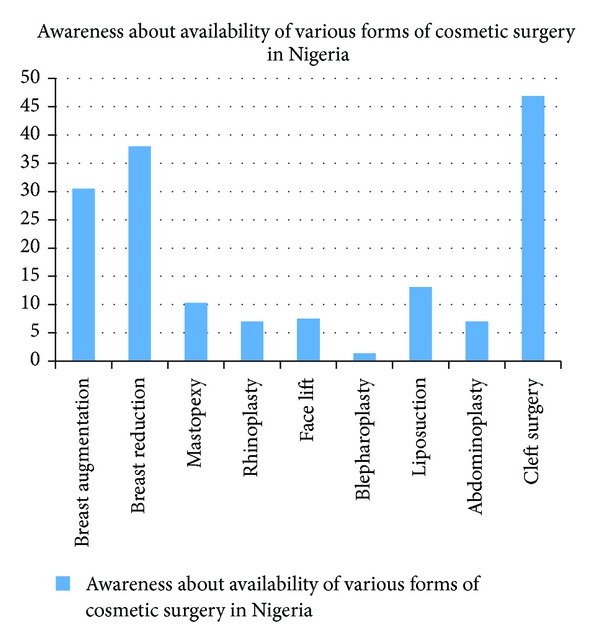
Suggested available cosmetic surgical procedures in Nigeria by respondents.

Breast reduction (67.1%), breast augmentation (61.0%), and cleft surgery (55.9%) ranked the highest as the commonest cosmetic surgeries respondents were aware of. However, the awareness about other forms of cosmetic surgeries included mastopexy (18.3%), rhinoplasty (34.7%), face lifts (44.6%), blepharoplasty (12.7%), liposuction (41.3%), and abdominoplasty, (31.5%) (Figure 2). Reduction (38.0%) and breast augmentation (30.5%) were the commonest forms of cosmetic surgeries perceived by the respondents as being practiced in Nigeria. Others were as shown in Figure 3.

One hundred and three (48.3%) of the respondents feel that the price of cosmetic surgery is above *₦*50, 000 ($326). Up to 83.1% of respondents feel that there are various forms of risks associated with cosmetic surgery which include deformation of body parts (20.2%), cancer (25.8%), keloid (40.4%), death (43.2%), infection (4.7%), and bleeding (2.3%). About 25.8% of respondents feel that the risk associated with cosmetic surgery is greater than other surgical procedures.

A good number of respondents 101 (47.4%) rated the Nigerian facility to be average with about 147 (69.0%) preferring facilities abroad over Nigerian facilities. One hundred and twenty-six (59.2%) of them feel that the outcome of cosmetic surgeries done outside Nigeria is better than that done in Nigeria.

One hundred and thirty-nine (65.3%) of the respondents considered cosmetic surgery necessary while 103 (60.1%) were ready to advice close relations or associates to go for cosmetic surgery of their choice when need arises. Of all the itemized cosmetic surgery, only cleft surgery was considered a necessary cosmetic surgery by 117 (54.9%) of the respondents.

If cosmetic surgeries were to be done free, only 43 (20.2%) of the respondents would go for cosmetic surgery of their choice. Seventy-six (35.7%) of the respondents felt that people's disposition will change towards them if they went for any cosmetic surgery.

Seventy-four (34.7%) of the respondents consider cosmetic surgery socially acceptable in Nigeria while 64 (30.0%) of them thought it was averagely acceptable. However, 94 (44.1%) and 128 (60.1%) of respondents considered cities and megacities, respectively, as environments where cosmetic surgeries were more acceptable. One hundred and seventy-five (82.2%) felt that cosmetic surgery was more acceptable with those in high socioeconomic class while 142 (66.7%) felt that they were more acceptable to the literates.

Awareness about cosmetic surgery was considered low by 89   (41.8%) of the respondents while 47 (22.1%) of the respondents considered awareness of cosmetic surgery as very low. One hundred and forty-nine (70.0%) of the respondents recommended the need for more awareness of cosmetic surgery in the society.

When considering their religious beliefs and cosmetic surgery, 85 (39.9%) of respondents considered cosmetic surgery to be godly and 175 (82.2%) of respondents were not aware of any beliefs against or taboo about cosmetic surgery.

There is significant statistical relationship between the occupation of respondents and their awareness about cosmetic surgery (*P* = 0.000); however, there is no relationship between their awareness and their readiness to go for cosmetic surgery (*P* = 0.877). There is no statistical relationship between the reliability of the information about cosmetic surgery and their readiness to go for cosmetic surgery of their choice (*P* = 0.368). There is a statistical relationship between the religious background of our respondents and their awareness about cosmetic surgery (*P* = 0.000). Also the estimation of Nigerian facilities showed statistical relationship with their preference for facilities outside Nigeria (*P* < 0.000). Sex shows no statistical relationship with the choice of going for cosmetic surgery (*P* > 0.329); however, females (11.3%) are more likely to consider cosmetic surgery than men (8.9%). This study reported a positive association between openness and choice of going for cosmetic surgery (*P* < 0.000).

## 4. Discussion

People in today's world are more health conscious, and awareness of the different medical specialties is on the increase. Despite the tremendous advancements in the field of plastic surgery, there seems to be a limited knowledge among the general public and also among medical professionals regarding the spectrum of plastic surgery. As a medical specialty, plastic surgery is poorly understood by both the general public and some medical professionals as well. In this study, the level of awareness was noted to be about 94.4%. However, there are varying levels of awareness about various forms of cosmetic surgery. In a similar study done in India, 12% of participants felt that plastic surgery and cosmetic surgery are the same, and 80% felt that cosmetic surgery is a part of plastic surgery. Of the 100 participants, 83% did not know why plastic surgery is called plastic surgery, 5% felt that it is called plastic surgery because it involves use of plastic, and 4% felt that it is called plastic surgery because face looks shiny like plastic after the surgery [9].

Information about cosmetic surgery among the respondents showed that more than half (126 (59.2%)) of the respondents got their information on cosmetic surgery from television and radio. This suggests the role of mass media in educating the public and disseminating information about cosmetic surgery. In a similar study done in India, nurses got their information mainly from television or magazines, although 27% of nurses gained the information through work; medical students learned about plastic surgery from all sources, but television and magazines were the main source [9].

In the Indian study [10], 37% of them believed that plastic surgery is an expensive surgery and meant for the rich and the famous. This is similar to what was obtained among the respondents in the current study who felt that cosmetic surgery is expensive and that cities and megacities were the environment where cosmetic surgeries are more acceptable among the high social economic class.

This study reported a positive association between openness and choice of going for cosmetic surgery, although previous work has reported a similar positive association between openness and positive self-evaluations of appearance [11].

Our results showed that women were more likely than men to consider having cosmetic surgery, which is consistent with previous work in which participants were asked to rate their likelihood of having various cosmetic procedures [12–15]. As discussed elsewhere, this sex difference may reflect the greater sociocultural pressure that women experience to live up to idealized images of physical perfection [5, 6].

Although 94% of the respondents in this study were aware of cosmetic surgery, 41.8% of them believed that awareness of cosmetic surgery was low (41.8%) while 22.1% of them considered the awareness as very low. This may mean that the respondents believed that the awareness of cosmetic surgery is low among the general public. Indeed 70% of them recommend the need to raise more awareness about cosmetic surgery in the environment. Since television and radio were the major sources through which majority of the respondents knew about cosmetic surgery, these sources will serve as a good source of enlightening others in the public. Health talks on cosmetic surgery, debates, and question and answers on the radio and television about these developing areas of plastic surgery will assist in educating the public and correcting any misgivings on cosmetic surgery.

It should be noted that 139 (65.3%) considered cosmetic surgery necessary while 128 (60.1%) were ready to advice close relations or associates to go for cosmetic surgery of their choice when need arose. However, cleft surgery seemed to be the only form of cosmetic surgery considered necessary by 117 (54.9%) of the respondents. This quietly suggests an unannounced turning down of other forms of cosmetic surgeries and thus low acceptance.

It should also be noted that no particular religious or cultural taboo or belief was noted against cosmetic surgery.

## 5. Conclusion

The awareness and disposition of health workers to cosmetic surgery are still low in developing countries like Nigeria. This is evidenced from the fact that the awareness and disposition among health care workers in a tertiary institution are rather low and many would prefer treatment outside the country when there is a need for such treatment overtreatment in the country. The health care workers are the first source of education and raising of awareness of any health procedures among the populace. The low level of this group of people therefore attests to the possible lower level still among the populace. Despite the rapid progress that has occurred in the field of plastic surgery, a large portion of the population is still unaware of the specialty. Therefore, they may not be taking advantage of the optimal care that is already available. If patients are to receive the best treatment available, it is essential to institute programs to educate healthcare consumers and providers about plastic surgery and its different subspecialties, especially the cosmetic procedures and their role within the healthcare system.

## Figures and Tables

**Table 1:** Distributions of respondents' sociodemographic data (*n* = 213).

Variable	Frequency (*n*)	Percentage (%)
Age group		
18–30 years	176	82.6
31–40 years	28	13.1
41–50 years	7	3.3
51–60 years	2	0.9
Sex		
Male	78	36.6
Female	135	63.4
Religion		
Christianity	179	84.1
Islam	33	15.5
Traditional	1	0.5
Occupation		
Medical student	79	37.1
Nursing student	60	28.2
Doctor	33	15.5
Nurse	32	15.0
Administrative worker	4	1.9
Pharmacist	1	0.5
Ward maid	4	1.9

## Appendix

### Questionnaire: Awareness and Attitude of Health Workers to Cosmetic Surgery in Osogbo, Nigeria

This questionnaire was designed to obtain information concerning your awareness and attitude concerning cosmetic surgery. Please note that your participation is voluntary and that the information given will be treated as confidential and anonymous. Please sign below if you understand the details information given about the study and you are willing to participate in the study.

*Section 1*:* Sociodemographic Factors*

1. Age …
2. Sex:
  [  ] Male
  [  ] Female
3. Occupational Status:
  [  ] Medical Student
  [  ] Nursing Student
  [  ] Medical Lab Scientist
  [  ] Doctor
  [  ] Nurse
  [  ] Administrative
  [  ] Pharmacist
  [  ] Ward Maid
4. Religious Background
  [  ] Christianity
  [  ] Islam
  [  ] Traditional

*Section 2: Knowledge and Awareness about Cosmetic Surgery*. Tick an appropriate response from the following, that is, “yes”, “no”, or “not sure”, except where other options are given:

(5) Are you aware of cosmetic surgery?
  [  ] Yes
  [  ] No
  [  ] Not sure
(6) If (6) is yes, which of the following informed you?
  [  ] Tv.
  [  ] radio
  [  ] friend
  [  ] posters
  [  ] medical consultation
  [  ] medical text book
  [  ] internet
(7) Did you consider the information reliable?
  [  ] Yes
  [  ] No
  [  ] Not sure
(8) Which of the following do you think do cosmetic surgery?
  [  ] general surgeon
  [  ] Maxillofacial surgeon
  [  ] plastic surgeon
  [  ] Orthopaedic surgeon
  [  ] Urology
(9) Which of the following Cosmetic Surgery are you aware of?
  [  ] breast augmentation
  [  ] breast reduction
  [  ] Mastopexy
  [  ] Rhinoplasty
  [  ] Face lift
  [  ] Blepharoplasty
  [  ] Liposuction
  [  ] Abdominoplasty
  [  ] Cleft Surgery
(10) Do you know if cosmetic surgery is done in Nigeria?
  [  ] Yes
  [  ] No
  [  ] Not sure
(11) If yes, which one(s) is/are done in Nigeria?
  [  ] Breast Augmentation
  [  ] Breast Reduction
  [  ] Mastopexy
  [  ] Rhinoplasty
  [  ] Face lift
  [  ] Blepharoplasty
  [  ] Liposuction
  [  ] Abdominoplasty
  [  ] Cleft Surgery
(12) What do you think is the price range for cosmetic surgery in Nigeria?
  [  ] 10,000–20,000
  [  ] 25,000–40,000
  [  ] 50,000–100,000
  [  ] >100,000
(13) Do you have any relative or friend who has undergone cosmetic surgery before?
  [  ] Yes
  [  ] No
  [  ] Not sure
(14) If yes, which type of cosmetic surgery?
  [  ] Breast Augmentation
  [  ] Breast Reduction
  [  ] Mastopexy
  [  ] Rhinoplasty
  [  ] Face lift
  [  ] Blepharoplasty
  [  ] Liposuction
  [  ] Abdominoplasty
  [  ] Cleft Surgery
(15) Do you know of any risk associated with Cosmetic surgeries?
  [  ] Yes
  [  ] No
  [  ] Not sure
(16) If yes, which kind of risk are you aware of?
  [  ] Deformation of body parts
  [  ] Cancer
  [  ] Keloid
  [  ] Death
  [  ] others, (please specify)…
(17) Do you think the risk of cosmetic procedure is greater than other surgical procedures?
  [  ] Yes
  [  ] No
  [  ] Not sure
(18) Are you aware of any facilities where Cosmetic Surgery is done in your locality?
  [  ] Yes
  [  ] No
  [  ] Not sure
(19) If (18) is yes, mention the facilities you know…
(20) You would like to rate the facilities for Cosmetic Surgery in Nigeria as?
  [  ] Excellent
  [  ] Good
  [  ] Average
  [  ] Bad

*Section 3: Attitude and Disposition to Cosmetic Surgery*

(21) If you were to make a choice, will you chose Nigerian facilities over facilities abroad for cosmetic surgery?
  [  ] Yes
  [  ] No
  [  ] Not sure
(22) Do you think the complications associated with cosmetic surgeries done in Nigeria differ from the ones done outside the country?
  [  ] Yes
  [  ] No
  [  ] Not sure
(23) If (22) is yes, which of the following do you think it is due to?
  [  ] Lack of facilities/equipment
  [  ] Lack of Modern operative techniques
  [  ] Lack of adequate pre/peri/post-operative care
  [  ] Inexperienced personnel
  [  ] Others (specify)…
(24) Do you think the outcome of cosmetic surgeries done in Nigeria and outside the country differ?
  [  ] Yes
  [  ] No
  [  ] Not sure
(25) Would you advice any close relative of yours to do cosmetic surgery when he/she needs one?
  [  ] Yes
  [  ] No
  [  ] Not sure
(26) Do you think Cosmetic Surgeries are necessary at all?
  [  ] Yes
  [  ] No
  [  ] Not sure
(27) If yes, which one(s) of these do you think is/are necessary?
  [  ] Breast Augmentation
  [  ] Breast Reduction
  [  ] Mastopexy
  [  ] Rhinoplasty
  [  ] Face lift
  [  ] Blepharoplasty
  [  ] Liposuction
  [  ] Abdominoplasty
  [  ] Cleft Surgery
(28) If cosmetic surgeries were done free, would you go for any Cosmetic surgery of your choice?
  [  ] Yes
  [  ] No
  [  ] Not sure
(29) Do you think it is godly to go for cosmetic surgery?
  [  ] Yes
  [  ] No
  [  ] Not sure
(30) Do you think people's disposition will change about you if you go for cosmetic surgery?
  [  ] Yes
  [  ] No
  [  ] Not sure
(31) If you were aware that someone did cosmetic surgery, will it negatively affect your relationship with such a person?
  [  ] Yes
  [  ] No
  [  ] Not sure
(32) Would you be open with the information that you have done Cosmetic Surgery before if you did one?
  [  ] Yes
  [  ] No
  [  ] Not sure
(33) Do you consider Cosmetic Surgery as socially acceptable in Nigeria?
  [  ] Yes
  [  ] No
  [  ] Not sure
(34) If (33) is yes, to what extent do you think it is acceptable?
  [  ] Widely acceptable
  [  ] Averagely acceptable
  [  ] Not acceptable
(35) If (33) is no, which of the following do you think affects the acceptability of cosmetic surgeries?
  [  ] Societal taboos
  [  ] Awareness
  [  ] Knowledge
  [  ] Exposure
  [  ] Environment
  [  ] Others, specify…
(36) In which type of Nigerian environment do you think cosmetic surgery is more acceptable?
  [  ] Villages
  [  ] Towns
  [  ] Cities
  [  ] Megacities
(37) In which group of the society do you think cosmetic surgery is more acceptable?
  *Economic Class*
    [  ] Low socioeconomic Class
    [  ] Middle Class
    [  ] Lower Middle
    [  ] Upper Middle
    [  ] High Class
  *Level of Education*
    [  ] Literate
    [  ] Illiterate
(38) Do you know of any taboo that is against cosmetic surgeries?
  [  ] Yes
  [  ] No
  [  ] Not sure
(39) If (38) is yes, specify…
(40) Would you like that more awareness program on cosmetic surgery be done?
  [  ] Yes
  [  ] No
  [  ] Not sure
(41) On what platform will you recommend the awareness to be done? Please specify…

Signature/Thumbprint: …

Date: …
